# Transcriptome analysis reveals the molecular mechanisms underlying growth superiority in a novel grouper hybrid (*Epinephelus fuscogutatus*♀ × *E. lanceolatus*♂)

**DOI:** 10.1186/s12863-016-0328-y

**Published:** 2016-01-19

**Authors:** Ying Sun, Chuan-Yu Guo, Deng-Dong Wang, Xiao Feng Li, Ling Xiao, Xinhui Zhang, Xinxin You, Qiong Shi, Guo-Jun Hu, Chao Fang, Hao-Ran Lin, Yong Zhang

**Affiliations:** State Key Laboratory of Biocontrol, Institute of Aquatic Economic Animals and Guangdong Provincial Key Laboratory for Aquatic Economic Animals, School of Life Sciences, Sun Yat-Sen University, Guangzhou, 510275 China; Shenzhen Key Lab of Marine Genomics, BGI, Shenzhen, 518083 China; Guangdong Provincial Key Lab of Molecular Breeding in Marine Economic Animals, Shenzhen, 518083 China

**Keywords:** Transcriptome, Growth superiority, GH/IGF axis, Hybrid grouper

## Abstract

**Background:**

Groupers (*Epinephelus* spp.) have been widely cultivated in China and South-East Asian countries. As a novel hybrid offspring crossed between *E. fuscogutatus*♀ and *E. lanceolatus*♂, Hulong grouper exhibits significant growth superiority over its female parent, which made it a promising farmed species in grouper aquaculture industry in China. Hulong grouper present a good combination of beneficial traits from both parent species, but the molecular mechanisms of its heterosis still remain poorly understood.

**Results:**

Based on RNA sequencing and gene expression profiling, we conducted comparative transcriptome analyses between Hulong grouper and its parents *E. fuscoguttatus & E. lanceolatus*. Six hundred sixty-two and 5239 differentially expressed genes (DEGs) were identified in the brains and livers, respectively. GO enrichment analysis of these DEGs revealed that metabolic process and catalytic activity were the most enriched GO terms. Further analysis showed the expressions of *GnRH1*and *GnRH3* in the brain, and GH/IGF axis related genes such as *IGF-1*, *IGF-2b*, *IGFBP-1*, *IGFBP-2*, *IGFBP-4* and *IGFBP-5a* in the liver of the hybrid F1 were significantly up-regulated, which is in accordance with the growth superiority of hybrid grouper. Meanwhile, expressions of genes related to the protein and glycogen synthesis pathway, such as *PI3KC*, *PI3KR*, *Raptor*, *EIF4E3*, and *PP1* were up-regulated, while *PYG* expression was down-regulated. These changes might contribute to increased protein and glycogen synthesis in the hybrid grouper.

**Conclusions:**

We identified a number of differentially expressed genes such as *GnRH1* and *GnRH3*, and genes involved in GH/IGF axis and its downstream signaling pathways for protein and glycogen synthesis in Hulong Grouper. These findings provided molecular basis underlying growth superiority of hybrid grouper, and comprehensive insights into better understanding the molecular mechanisms and regulative pathways regulating heterosis in fish.

**Electronic supplementary material:**

The online version of this article (doi:10.1186/s12863-016-0328-y) contains supplementary material, which is available to authorized users.

## Background

As economically important fish species in marine aquaculture, groupers (*Epinephelus* spp.) are known for their delicious taste, tender flesh and rich nutrition [1]. In the past decades, grouper industry has developed rapidly and many grouper species have been widely cultivated in China and South-East Asian countries [2, 3]. However, the sustainable development of grouper industry has been threatened by the degradation of germplasm resources and availability of grouper fries in hatcheries [4]. To resolve these problems, hybridization technology has been introduced into grouper artificial breeding.

Hybridization is defined as a successful mating strategy for two species with one or more heritable traits, and is often used in artificial breeding to obtain potentially desirable traits in their offsprings [5, 6]. In grouper species, hybridization was firstly achieved between white-spotted green grouper (*E. amblycephalus*) and red grouper (*E. akaara*) [7]. From then on, more efforts have been made in developing hybrid groupers and about eight hybrids with various favorable traits, such as faster growth and development, stronger immunity and higher survival rate were obtained [8–12].

Brown-marbled grouper (*E. fuscoguttatus,* Efu) and Giant grouper (*E. lanceolatus,* Ela) are both important breeding fishes with different growth rates and disease resistance. The former is a slow growing but long-lived species with high disease resistance [13, 14], while Giant grouper is popular breeding species for its rapid growth, reaching up to 3 kg in the first year [15]. Previously, by fertilizing the eggs of Brown-marbled groupers with the sperms of Giant grouper, we obtained a novel hybrid grouper, Hulong grouper (Hyb). It was shown that Hulong grouper combines physiological features of both parental species [16], and its growth rates is 35.9 % faster than that of the maternal Brown-spotted grouper [17]. According to our preliminary data of growth (Zhang Y. et al., unpublished data), at the age of 18 months, the hybrid Grouper (Hyb) reaches to 1174.7 ± 264.2 g (*n* = 10), while the paternal Grouper (Ela) is only 838.1 ± 168.2 g (*n* = 15). Although the sample size was not large enough, the overall result was statistically significant and demonstrated that the hybrid Grouper (Hyb) grows faster than the paternal Grouper (Ela) at the age of 18 months. In recent years, many researches have examined their biological and physiological features of the hybrid groupers, however, very little is known about its genetic mechanisms of heterosis.

High-throughput RNA sequencing technology (RNA-Seq) has been used as an effective tool for transcriptome analysis, aiming to discover, profile and quantify RNA transcripts [18]. RNA-Seq combines the advantages of microarray and EST-sequencing, including single-base resolution, high throughput, low background noise, and high sensitivity, which make it feasible for mapping of transcribed regions, quantification of gene expression levels, and distinction of different isoforms and allelic expression [18–22]. In recently decades, RNA-Seq has been widely used to identify differential genes expressions related to heterosis in crop plants and cultured fishes [23–26].

In teleosts, growth hormone/insulin-like growth factor (GH/IGF) axis, including the effector hormones GH, IGFs (IGF-1 and IGF-2) and a regulatory feedback loop between them, plays an important role in regulating somatic growth [27, 28]. GH is a pituitary hormone regulating various physiological processes such as somatic growth, behavior, immune function, lipid and protein metabolism, osmoregulation, and feeding behavior in fishes [27, 29, 30]. It was widely acknowledged that GH stimulates hepatic and circulating IGF-1 levels in teleosts [31, 32], whereas IGF system, consisting of IGFs (IGF-1 and IGF-2), cell surface receptors (IGF-1R and IGF-2R) and IGF-binding proteins (IGFBP-1 to −6), plays a crucial role in cell growth, proliferation and differentiation [33]. Among them, IGF-1 is positively correlated with growth rate and IGF-1 levels in serum is served as a growth index in fishes [34, 35], as demonstrated in Nile tilapia (*Oreochromis niloticus*) [36], Mozambique tilapia (*Oreochromis mossambicus*) [37] and Mud carp (*Cirrhinus molitorella*) [38].

In our study, comparative transcriptomic analysis for hybrid grouper and its parents Efu and Ela was conducted to explore the molecular basis underlying the growth superiority of hybrid grouper. The brain is an important organ that centralized control over the other organs of the body by driving secretion of various hormones to regulate the growth of soma. The liver is also critical for its major roles in metabolism with numerous functions in the fish, including regulation of glycogen storage, plasma protein synthesis, and hormone production. Therefore, the brain and liver were selected as the genetic mechanism targets. Our results provided comprehensive data with respect to differential gene expressions in the GH-IGF pathway and its downstream signaling pathways involved in the heterosis of the hybrid grouper. This will contribute towards improving our understanding of the molecular mechanisms and regulative pathways regulating heterosis in fish.

## Results

### Transcriptome sequencing and alignment to the reference grouper genome

To provide transcriptome profiling of the hybrid F1 and its parents, brain and liver cDNA libraries from the three groupers (Hyb and its parents Efu & Ela) were separately prepared and subjected to RNA-Seq analysis in BGI-Shenzhen. In total, 323 million high-quality clean reads were obtained after data filtering (Table 1). Subsequently, the clean reads were aligned to the *E. coioides* genome (Zhang Y. et al., unpublished data) using SOAP aligner 2.0 [39]. About 46–61 % of reads were mapped to the reference genome (≤5 base mismatches), in which 28–41 % of reads were mapped to the gene regions (Table 1). In addition, 28–40 % of those uniquely mapped reads were further used for gene quantification analysis (Table 1). The species of reference genome is orange-spotted grouper (*E. coioides*), which is different from the hybrid grouper and its two parents. The above-mentioned low mapping rates could mainly result from the species differences.

**Table 1:** Summary of transcriptome data generated from hybrid F1 Hulong and its parents

Sample	Number of clean reads	Number of reads mapped to genome	Mapping rate	Number of reads mapped to genes	Mapping rate	Unique match to genes	Mapping rate
			(≤5 base mismatches)		(≤5 base mismatches)		
Ela-Brain	52,132,944	31,936,098	61.26 %	16,517,993	31.68 %	16,267,989	31.20 %
Ela-Liver	54,047,848	27,007,104	49.97 %	21,377,674	39.55 %	20,869,693	38.61 %
Hyb-Brain	53,658,808	32,293,926	60.18 %	15,630,940	29.13 %	15,390,675	28.68 %
Hyb-Liver	54,743,866	26,992,311	49.31 %	22,217,145	40.58 %	21,718,613	39.67 %
Efu-Brain	54,079,346	31,403,025	58.07 %	15,452,191	28.57 %	15,215,841	28.14 %
Efu-liver	54,930,262	25,223,125	45.92 %	20,932,386	38.11 %	20,366,111	37.08 %

Ela, Hyb, and Efu denote *Epinephelus lanceolatus* ♂, Hybrid F1, and *Epinephelus fuscogutatus* ♀, respectively

### DEGs among the hybrid F1 and its parents *Efu & Ela*

**Fig. 1:**
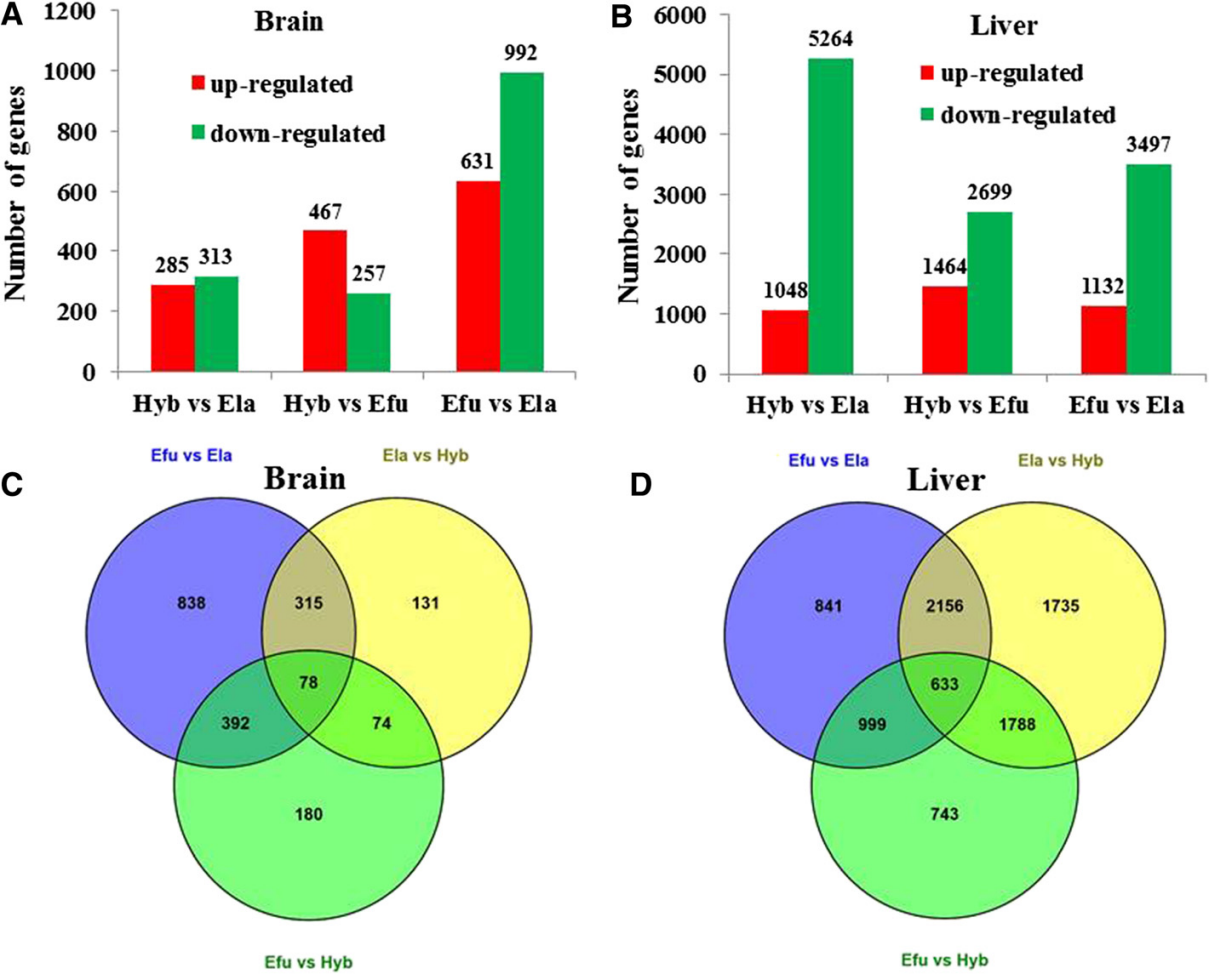
DEGs and the overlaps of DEGs among parental *E. fuscoguttatus* (Efu), *E. lanceolatus* (Ela) and their hybrid offsprings (Hyb). Different numbers of DEGs in brains (**a**) and livers (**b**) were identified. The overlaps of DGEs in the brain (**c**) and the liver (**d**) were also determined

RPKMs (Reads Per Kilobase Transcriptome per Million mapped reads) were used to quantify gene expression levels. The RPKM values of each gene in the brain and liver tissues of the hybrid grouper were compared with those in its parents Efu & Ela, respectively (Additional file 1). Through filtering by the criteria that false discovery rate (FDR) ≤0.05 and absolute log2 (ratio) ≥1, we identified DEGs between the hybrid grouper and its parents (Fig. 1a and b) (Additional files 2, 3, 4, 5). In the brain, there were 285 and 467 up-regulated genes in the Hyb compared to Ela and Efu, respectively, and the corresponding numbers of down-regulated genes were 467 and 257, and a total of 631 up-regulated genes and 992 down-regulated genes were identified in Efu relative to Ela (Fig. 1a). In the liver, the Hyb had 5264 and 2699 down-regulated genes compared to Ela and Efu, respectively, and Ela had 3497 down-regulated genes relative to Efu (Fig. 1b). These results suggest more divergent distribution of gene expression exists in the livers than in the brains. We also determined whether the genes differentially expressed between the parents overlap the genes that differ between each parent and the hybrid. In the brain, the number of overlap DGEs between ‘Ela vs Efu’ and ‘Ela vs Hyb’ was 393, and the number of overlap DGEs between ‘Ela vs Efu’ and ‘Ela vs Hyb’ was 470 (Fig. 1c). In the liver, there were 4629 DGEs between the parents, including 2789 and 1632 overlap DGEs when the Hyb was compared to Ela and Efu, respectively (Fig. 1d). The number of DGEs among three species in the brain and liver was 78 and 633, respectively (Fig. 1c and d). The hierarchical clustering maps of these DGEs were presented in Additional file 6: Figure S1 and Additional file 7: Figure S2. In the brain, 35 and 52 DE genes in the hybrid show overdominance and underdominance, respectively. In the liver, there were 595 and 1508 DE genes expressed more highly or lowly in the hybrid than in either parent, respectively.

### GO enrichment of DEGs

Gene ontology (GO) annotation of genes was achieved by Blast2GO [40], and. 53,665 GO terms were found and then assigned to 9503 annotated genes in the reference genome. Among them, 13,244 terms is related to cellular components, 14,191 terms for molecular function and 26,230 terms for biological process (Additional file 8: Figure S3).

**Fig. 2:**
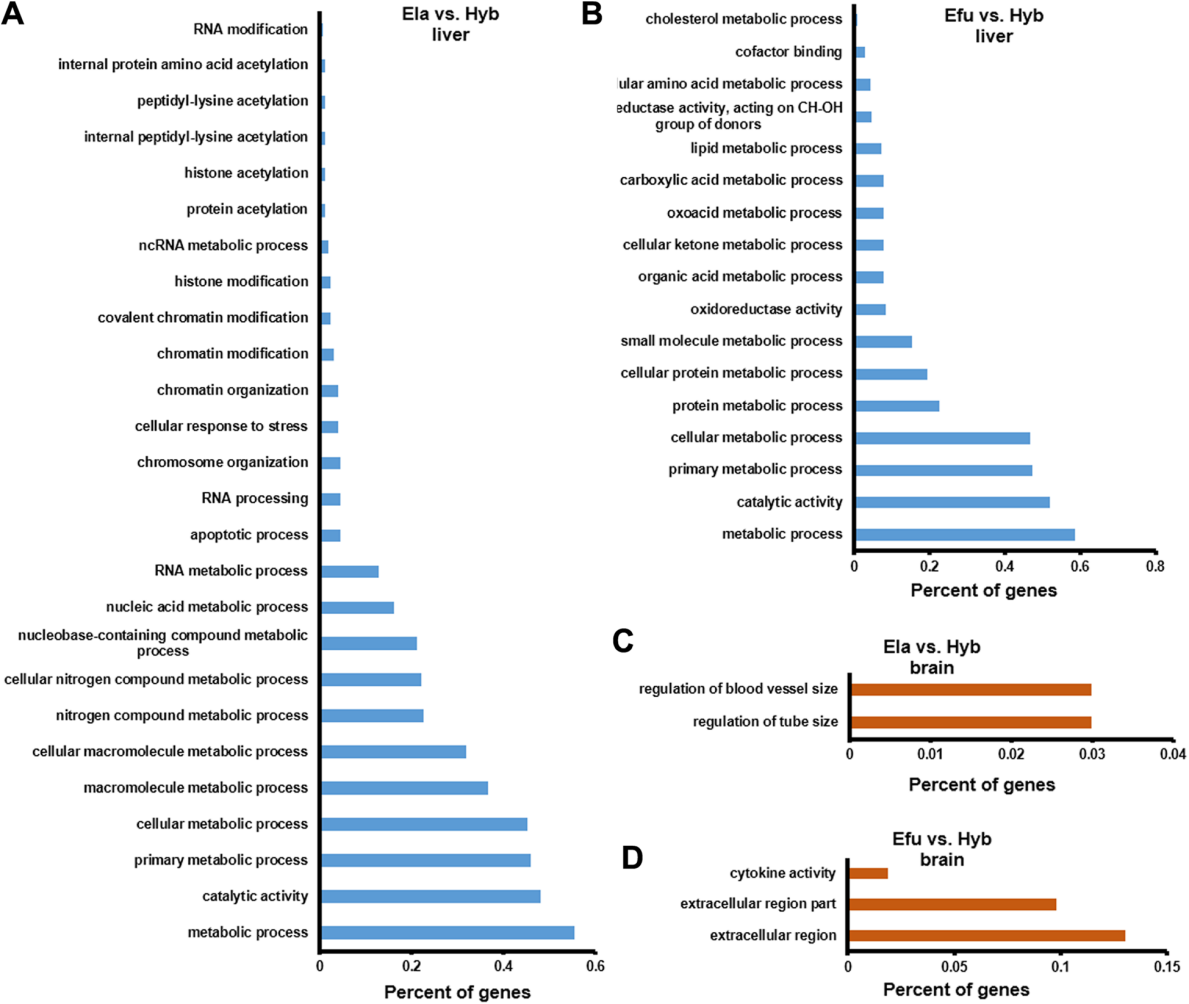
GO enrichment of DEGs in brains and livers of three fish species. **a** and **b**. GO biological process categories enrichment of DEGs in livers of three fish species. **c** and **d**. GO enrichment of DEGs in brains of three fish species. Compared with that in livers (**c** and **d**), the number of enriched GO terms in brain is less (**a** and **b**)

The identified DEGs were subsequently used for enrichment analysis by GO:Termfinder software using the hypergeometric test [41, 42], and *P*-values were corrected using Bonferroni method [43]. Being selected significantly enriched GO terms with Q-value < 0.05, the liver DEGs between Hyb and Ela, and between Hyb and Efu were enriched into 40 and 22 GO terms respectively, which provides an overview of ontology content (Additional files 9 and 10). In the molecular function and biological process categories, metabolic process (GO:0008152) and catalytic activity (GO:0003824) were the most enriched GO terms (Fig. 2a  and b), suggesting that extensive metabolic and catalytic activities in the liver might be associated with growth superiority of the hybrid grouper.

GO enrichment analysis was also performed for the brain DEGs between Hyb and its parents, and only 5 GO terms were enriched in the brain (Fig. 2c and d, Additional files 11 and 12), which is consistent with the fact that less number of DEGs were identified in brains than that in livers of these three grouper species.

### Comprehensive analysis of DEGs

**Fig. 3:**
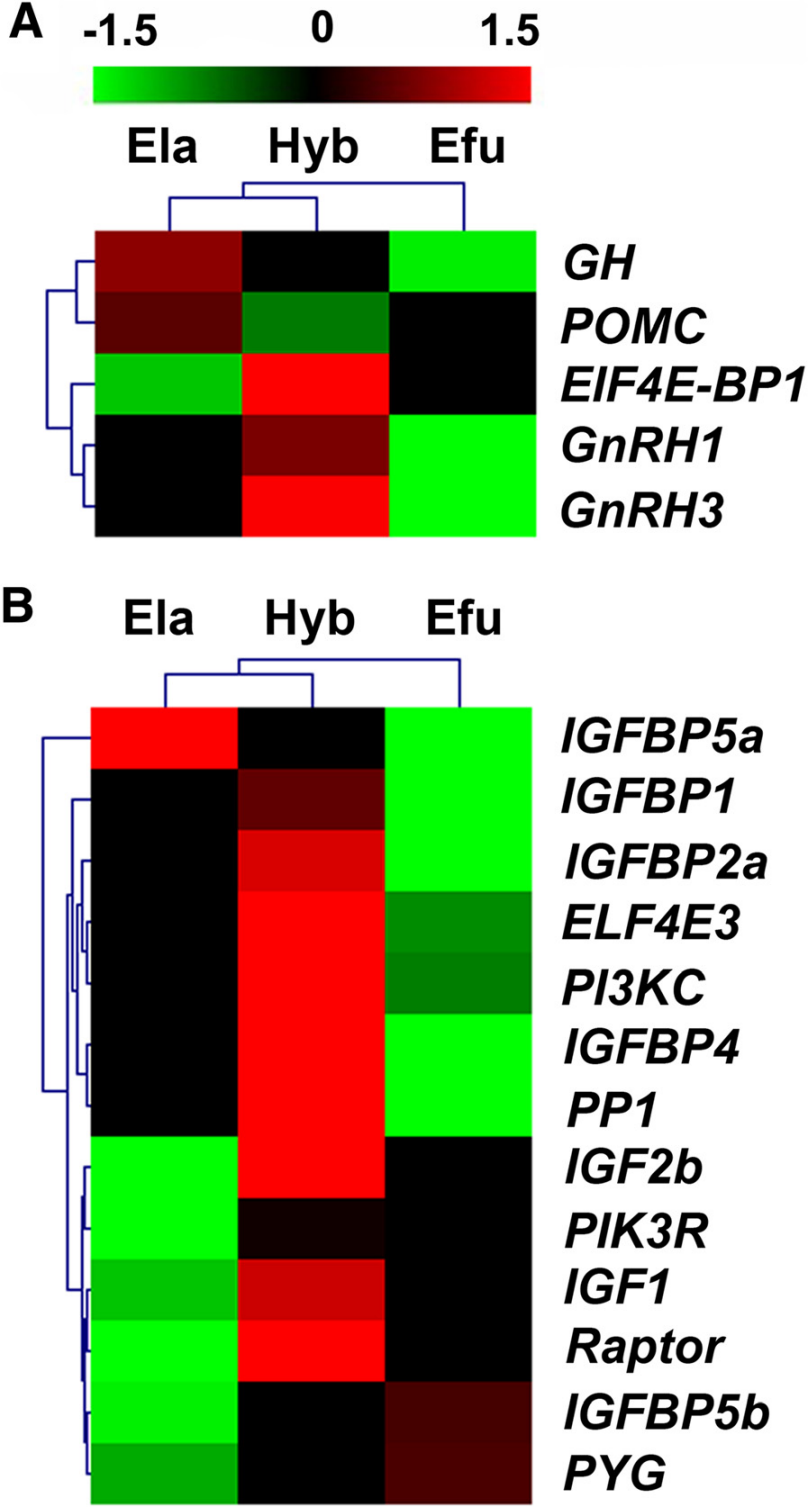
Hierarchical cluster analysis of DEGs involved in the GH/IGF and downstream pathways. The color key represents RPKM normalized log_2_ transformed counts in brains (**a**) and livers (**b**) of three fish species. Efu, Ela, and Hyb denote *E. fuscoguttatus, E. lanceolatus* and their hybrid F1, respectively

We further analyzed the DEGs in GH/IGF axis and its downstream signaling pathways, which is critical in the growth and development of fish. In brains, we found *GH* and the members of Gonadotropin-releasing hormone (*GnRH*), *GnRH1* and *GnRH3* were up-regulated in Hyb over the Efu (Fig. 3a). And genes in the GH/IGF axis, including *IGF-1*, *IGF-2b*, *IGFBP-1*, *IGFBP-2a*, *IGFBP-4*, *IGFBP-5a* and *IGFBP-5b* were differentially expressed in livers of the Hyb over its parents (Fig. 3b). In addition, the DEGs were identified in protein and glycogen synthesis signaling pathways, including *PI3K regulatory subunit* (*PI3KR*), *PI3K catalytic subunit* (*PI3KC*), *regulatory associated protein of mTOR* (*Raptor*), *eukaryotic translation initiation factor 4E* (*EIF4E3*), *protein phosphatase 1* (*PP1*), and *glycogen phosphorylase* (*PYG*) (Fig. 3b).

### Validation DEGs by quantitative real-time PCR (qRT-PCR)

**Fig. 4:**
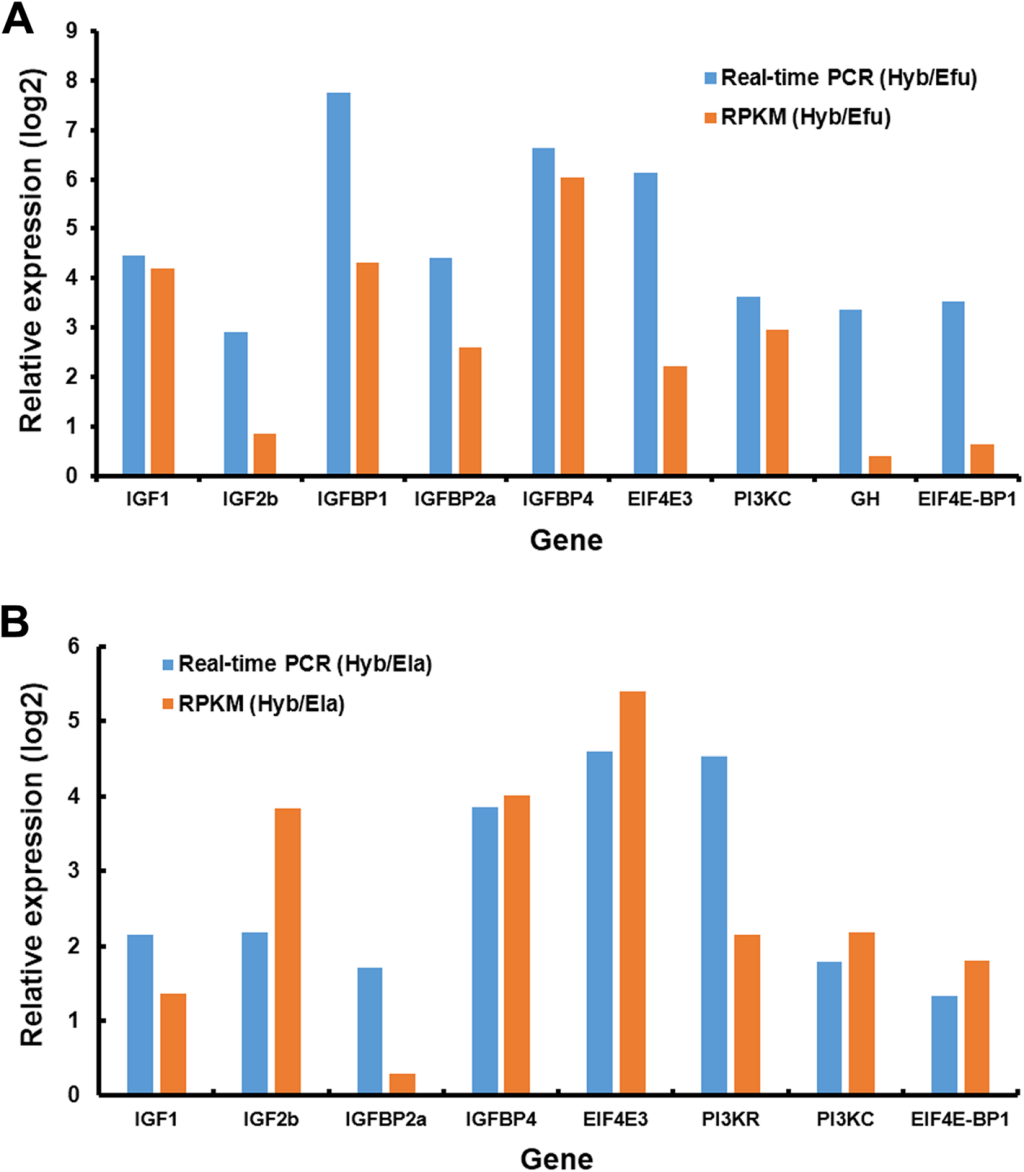
Validation of differentially expressed genes by Real-time PCR. β-actin was used as an internal control and each value represents average of three separate biological replicates. Real-time PCR validates the DEGs **a** between the Hyb and maternal Efu and **b** between the Hyb and paternal Ela. *GH* was detected in the brains, while other genes were examined in the livers

A subset of night important DEGs was randomly selected for qRT-PCR validation in livers and brains of the three grouper species, and the qRT-PCR primers were designed based on the mapped sequences (see the primer and sequence list in Additional file 13). The results were further compared with those generated from the transcriptome sequencing. Our results showed that the data from the two different methods are consistent (Fig. 4).

In summary, our data demonstrated that differential expression of *GnRH*, and genes related to GH/IGF axis and its downstream signaling pathways may be related to growth superiority in the hybrid grouper by increasing protein and glycogen synthesis. Our finding provides insights into molecular basis underlying the heterosis of hybrids in fish.

## Discussion

Hybridization is one of the most effective ways to improve physiological properties of species [44]. Over the years, many research efforts have been made in exploring the molecular basis of heterosis. The recent research progress includes the study on DEGs and its association with heterosis [25, 45]. For instance, 3488 DEGs were identified between the parental lines of rice and their F1 hybrids and further GO enrichment analysis of these DEGs revealed that metabolism categories were the most enriched terms, indicating metabolism pathway related DEGs might contribute to the heterosis of hybrid rice [45].

Here we conducted comparative transcriptomic study on hybrid Hulong and its parents Efu and Ela. 323 million high-quality reads were generated from the brains and livers of Hyb and its parental species. On average, 54.12 % of reads were mapped to the *E. coioides* genome (Zhang Y. et al., unpublished data), and 34.60 % of the reads were mapped to gene regions. Among the annotated transcripts, 15,107 DEGs in the liver and 2948 DEGs in the brain were identified, suggesting that the DEGs in the liver of Hulong grouper might play a more important role in its heterosis than those in its brain.

### DEGs associated with the GH/IGF axis

**Fig. 5:**
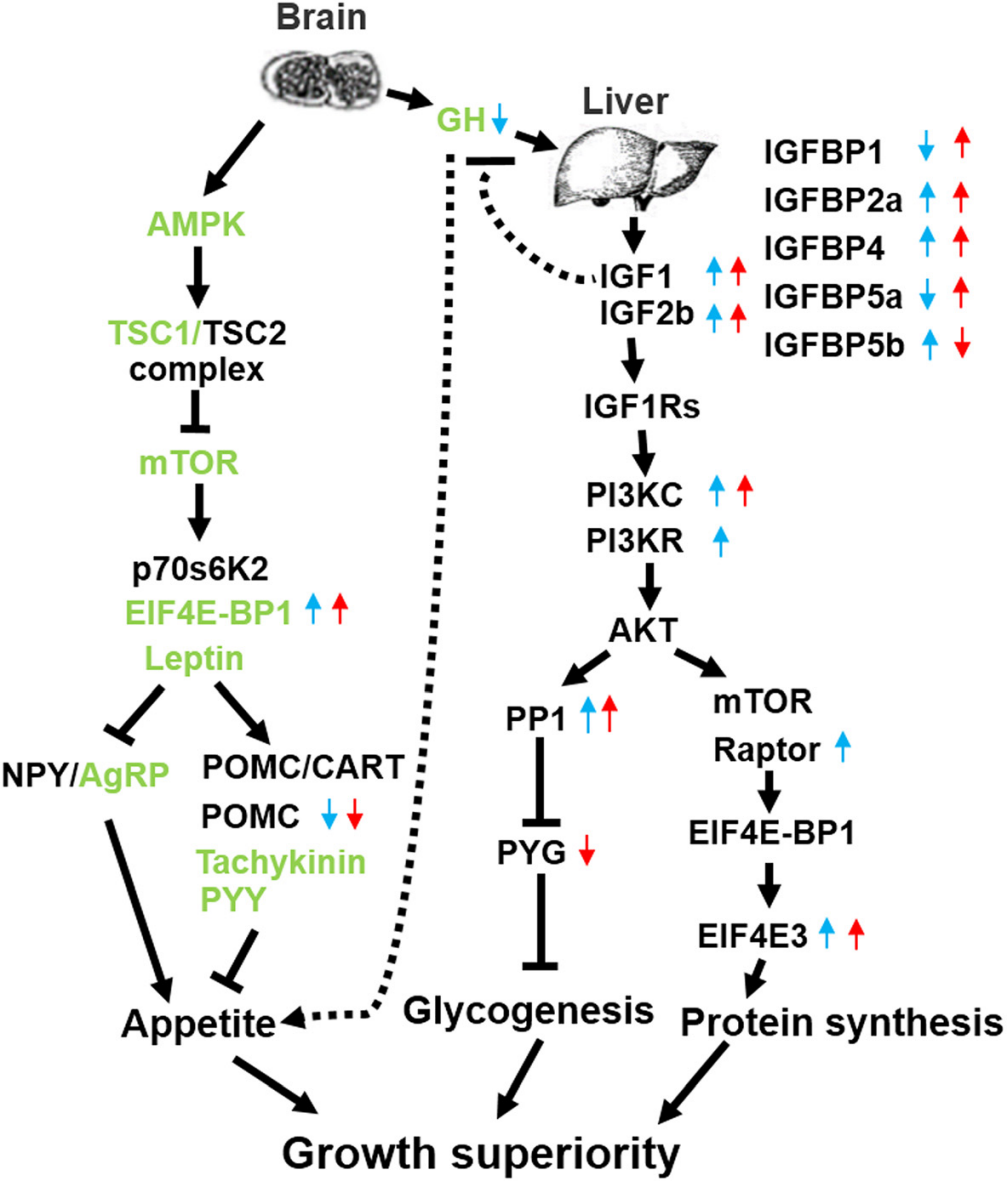
The predicted map of DEGs involved in the GH/IGF and downstream pathways regulated growth superiority. *Blue arrows* denote the genes with differential expression between the hybrid F1 and paternal *E. lanceolatus. Red arrows* denote the genes with differential expression between the hybrid F1 and maternal *E. fuscoguttatus. Up or down arrows* stand for up- or down-regulated in the hybrid compared with its parent(s)

The growth of vertebrates including fish is primarily regulated by the GH/IGF axis [27]. In present study, we found several growth-associated genes differentially expressed in the Hyb over its parents, such as *GnRH1*, *GnRH3*, and *GH* in the brain, and *IGF-1*, *IGF-2b*, *IGFBP-1*, *IGFBP-2a*, *IGFBP-4*, *IGFBP-5a* and *IGFBP-5b* in the liver (Fig. 5). Among them, the expression levels of *IGF-1* and *IGF-2b* in the hybrid F1 liver were significantly higher than those in their parents, indicating IGF-1 and IGF-2b might play a critical role in the growth of hybrid grouper. Although the expression of *GH* in the brain of hybrid grouper is only slightly higher than that in its paternal Ela, the possible explanation is that GH might be inhibited by the negative feedback from increased IGF-I level in the hybrid grouper [46].

Many neuroendocrinological factors such as GnRH1, GnRH3, pituitary adenylate cyclase activating polypeptide 1 (PACAP1), PACAP1 type 1a/b receptor (PACAP1ra/b1), thyrotropin releasing hormone (Trh), and dopamine receptor D1a (Drd1a) promote GH secretion in the brain [47–50]. Consistently, we found the gene expression level of *GnRH1* and *GnRH3* is higher in the hybrid grouper, compared with that in the maternal Efu , and the gene expression of *PACAP1*, *PACAPra/b1*, *Trh*, and *Dra1a* in the Hyb is slightly higher in Hyb than its maternal Efu.

IGFBPs were reported to bind IGFs with higher affinities than IGFR, leading to prevention of IGFs degradation, thereby prolonging the half-lives of IGFs in serum [51]. In the present study, we found the significant higher expression levels of *IGFBP-2a* and *IGFBP-4* in Hyb than those in its parents, indicating that IGFBP-4 and IGFBP-2 might be the dominant circulating binding proteins in groupers, as opposed to IGFBP3 in mammals [52]. In addition, our results indicated that the accumulation of *IGFBP-1*, *IGFBP-5a* and *IGFBP-5b* mRNAs in Hyb were intermediated between the two parents, while members of *IGFBP* family exhibited distinct expression patterns among these three fish species, demonstrating the independent physiological roles of its individual *IGFBP* family members [53]. Taken together, our results suggest that the differential gene expression related to GH/IGF axis might play an important role in the growth superiority of the hybrid grouper.

### DEGs involved in protein and glycogen synthesis

IGF-1 activates a series of phosphorylation events including PI3K/AKT pathway by binding its receptor IGFR1 [54], thus lead to many anabolic effects including protein synthesis [55] and glycogen synthesis [56]. Upon IGF-1 stimulation, PI3K/AKT activates mTOR [57], then Raptor is combined with mTOR to form mTORC1, which alters the activity of EIF4E-BP1 by phosphorylation [58]. The phosphorylated EIF4E-BP1 favors the dissociation of EIF4E from the inhibitory complex of EIF4E and EIF4E-BP1 [59]. Ultimately, the increased availability of EIF4E promotes the elevation of protein synthesis [59]. In our study, the higher expression level of *PI3KC*, *PI3KR*, *Raptor* and *EIF4E3* was observed in the hybrid F1 (Fig. 5), which suggests the strengthened capacity of protein synthesis in the hybrid grouper.

Another function of the PI3K/AKT pathway is to facilitate glycogen synthesis through generating activated PI3K and stimulating additional kinases, especially AKT (protein kinase B), and further activates protein PP1 (KEGG 04910) [60]. Subsequently, the activated PP1 inhibits PYG activity and then accelerates the glycogenesis process [61]. In line with this, our study showed that the expression of many genes involved in the PI3K/AKT pathway, such as *PI3KR*, *PI3KC*, and *PP1* were up-regulated in the liver of Hyb compared with that in the maternal Efu, while the expression levels of *PYG* in Hyb were lower than those in Efu, which potentially leads to the maintenance of the glycogenesis in Hyb.

## Conclusion

Using RNA-seq, we identified differentially expressed genes in Hulong grouper. GO enrichment results indicated that these genes were related to a variety of molecular functions, such as metabolic process and catalytic activity. Further analysis revealed that differential gene expression in GH/IGF axis and downstream signaling pathways might contribute to growth superiority of the hybrid grouper through enhancing protein synthesis and glycogen synthesis.

## Methods

### Fish and sample preparation

*E. fuscoguttatus* (♀), *E. lanceolatus* (♂) and their hybrid F1 (Hulong) were cultivated under same breeding conditions in Daya Bay Seawater Fish Farm in Huizhou, Guangdong Province, China. At the age of 18 months, three grouper species were obtained and accommodated under same natural conditions of food, water, light, and density for 3 days. Subsequently, three individuals from each group were randomly selected for further experiments. Fresh brain and liver tissues were respectively collected and immediately stored in liquid nitrogen. All experiments were performed in accordance with the guidelines of the Animal Ethics Committee and were approved by the Institutional Review Board on Bioethics and Biosafety of BGI.

### RNA extraction, libraries construction and high-throughput sequencing

Total RNA from the brain and liver tissues was extracted with Trizol reagent (Invitrogen, Carlsbad, CA, USA) and purified using RNeasy Animal Mini Kit (Qiagen, Valencia, CA) according to the manufacturer’s instructions. RNA Integrity Number (RIN) values were measured for all samples. Equal amount of total RNA from three fish individuals in each group were pooled and used to construct cDNA libraries for RNA sequencing.

The cDNA libraries were prepared following the manufacturer’s instructions (Illumina, San Diego, CA). In brief, the poly-A containing mRNA molecules was purified using poly-T oligo-attached magnetic beads. Subsequently, the mRNAs were fragmented into small pieces. The cleaved RNA fragments were copied into first strand cDNA using reverse transcriptase and random primers, and the second strand cDNA was synthesized using DNA polymerase I and RNase H. These cDNA fragments were ligated with the adapters, and these products were then purified and enriched with PCR to create the final cDNA libraries. Finally, the cDNA libraries were sequenced through Illumina HiSeq2000 system at BGI-tech (Shenzhen, China). Raw reads were submitted to SRA database in NCBI under accession number SRP056564.

### Processing of raw reads and quantification of differential gene expression levels

Raw reads were filtered by removing adaptors, reads with more than 5 % unknown nucleotides, and sequences shorter than 20 nt and low quality with Q <20. The resultant clean reads were mapped to the orange-spotted grouper (*E. coioides*) genome (Zhang Y. et al., unpublished data; using SOAP aligner 2.0 [39]), which has been *de novo* assembled at BGI-Shenzhen, China.

The quantification and differential expressed genes analyses were carried out by the Cufflink program 2.1.1. RPKMs (Reads Per Kilobase Transcriptome per Million mapped reads) were used to quantify the mapped whole gene expression levels. The differential expressed genes were determined through filtering by the criteria that false discovery rate (FDR) ≤0.05 and absolute log2 (ratio) ≥1.

### Validation of deep sequencing

Nine genes with significant differential expression among Hyb and its parents were selected randomly to validate the deep sequencing results. After purification of total RNA using SV Total RNA Isolation System (Promega, USA), reverse transcription reactions were performed to synthesize the cDNAs. Subsequent real-time PCRs were carried out using gene-specific primers (Additional file 10). β-actin was used as an internal control and the 2^-ΔΔCt^ method [62] was used to calculate relative expression amounts. All samples were examined in triplicate.

### Availability of supporting data

The data sets supporting the results of this article are included within the article and its additional files. The transcriptome reads produced in this study have been deposited in the National Center for Biotechnology Information (NCBI) SRA database with accession number of SRX974310 & SRX 974314, SRX 969045 & SRX974311, and SRX974309 & SRX974313 for the brain & the liver of the Hyb, Ela and Efu respectively. Access to the data is available upon publication at http://www.ncbi.nlm.nih.gov/sra/.

## Additional files

Additional file 1:
**RPKMs of all genes detected in the brains and livers of three grouper species.** (XLSX 4270 kb) Additional file 2:
**Differentially expressed genes between the brains of Hybrid F1 and maternal** ***E. fuscoguttatus.*** (XLSX 87 kb) Additional file 3:
**Differentially expressed genes between the brains of Hybrid F1 and paternal** ***E. lanceolatus.*** (XLSX 73 kb) Additional file 4:
**Differentially expressed genes between the livers of Hybrid F1 and maternal** ***E. fuscoguttatus.*** (XLSX 460 kb) Additional file 5:
**Differentially expressed genes between the livers of Hybrid F1 and paternal** ***E. lanceolatus.*** (XLSX 595 kb) Additional file 6: Figure S1. The hierarchical clustering map of DGEs among three species in the brain. Efu, Ela, and Hyb denote *E. fuscoguttatus, E. lanceolatus* and their hybrid F1, respectively. (PDF 40 kb) Additional file 7: Figure S2. The hierarchical clustering map of DGEs among three species in the liver. Efu, Ela, and Hyb denote *E. fuscoguttatus, E. lanceolatus* and their hybrid F1, respectively. (PDF 236 kb) Additional file 8: Figure S3.
**Functional annotation of grouper transcripts based on GO categorization.** The left y-axis indicates the percentage of a specific category of genes in that main category. The right y-axis indicates the number of genes in a category. (TIF 521 kb) Additional file 9:
**GO enrichment of DEGs between the livers of Hybrid F1 and maternal** ***E. fuscoguttatus.*** (XLSX 309 kb) Additional file 10:
**GO enrichment of DEGs between the livers of Hybrid F1 and paternal** ***E. lanceolatus.*** (XLSX 464 kb) Additional file 11:
**GO enrichment of DEGs between the brains of Hybrid F1 and maternal** ***E. fuscoguttatus.*** (XLSX 61 kb) Additional file 12:
**GO enrichment of DEGs between the brains of Hybrid F1 and paternal** ***E. lanceolatus.*** (XLSX 51 kb) Additional file 13:
**Sequences of the primers used for real-time RT-PCR and related gene sequences.** (XLSX 13 kb)

## Abbreviations

α-MSH: α-melanocyte stimulating hormone
AgRP: agouti-related peptide
AKT: protein kinase B
bp: base pair
cDNA: complementary DNA
CDS: coding sequences
DEGs: differentially expressed genes
Drd1a: dopamine receptor D1a
Efu: *E. fuscoguttatus*
Ela: *E. lanceolatus*
EIF4E3: eukaryotic translation initiation factor 4E
EIF4E-BP1: eukaryotic translation initiation factor 4E binding protein 1
EST: expressed sequence tags
FC: fold change
FDR: false discovery rate
GH: growth hormone
GnRH: gonadotropin-releasing hormone
GO: gene ontology
Hyb: hybrid
IGF: insulin-like growth factor
IGF-1R: IFG-1 receptor
IGFBP: IGF binding protein
PACAP1: pituitary adenylate cyclase activating polypeptide 1
PI3KC: phosphoinositide-3-kinase catalytic subunit
PI3KR: phosphoinositide-3-kinase regulatory subunit
PP1: protein phosphatase 1
PYG: glycogen phosphorylase
Raptor: regulatory associated protein of Mtor
RPKMs: reads per kilobase transcriptome per million mapped reads
Trh: thyrotropin releasing hormone
