# Supplementary material for: Transcriptome analysis reveals the molecular mechanisms underlying growth superiority in a novel grouper hybrid (Epinephelus fuscogutatus♀ × E. lanceolatus♂)
Source: BMC Genet. 2016 Jan 19;17:24. doi: 10.1186/s12863-016-0328-y (PMC4719697; doi:10.1186/s12863-016-0328-y)
Supplement: Additional file 6: Figure S1. — The hierarchical clustering map of DGEs among three species in the brain. Efu, Ela, and Hyb denote E. fuscoguttatus, E. lanceolatus and their hybrid F1, respectively. (PDF 40 kb) [file 12863_2016_328_MOESM6_ESM.pdf]

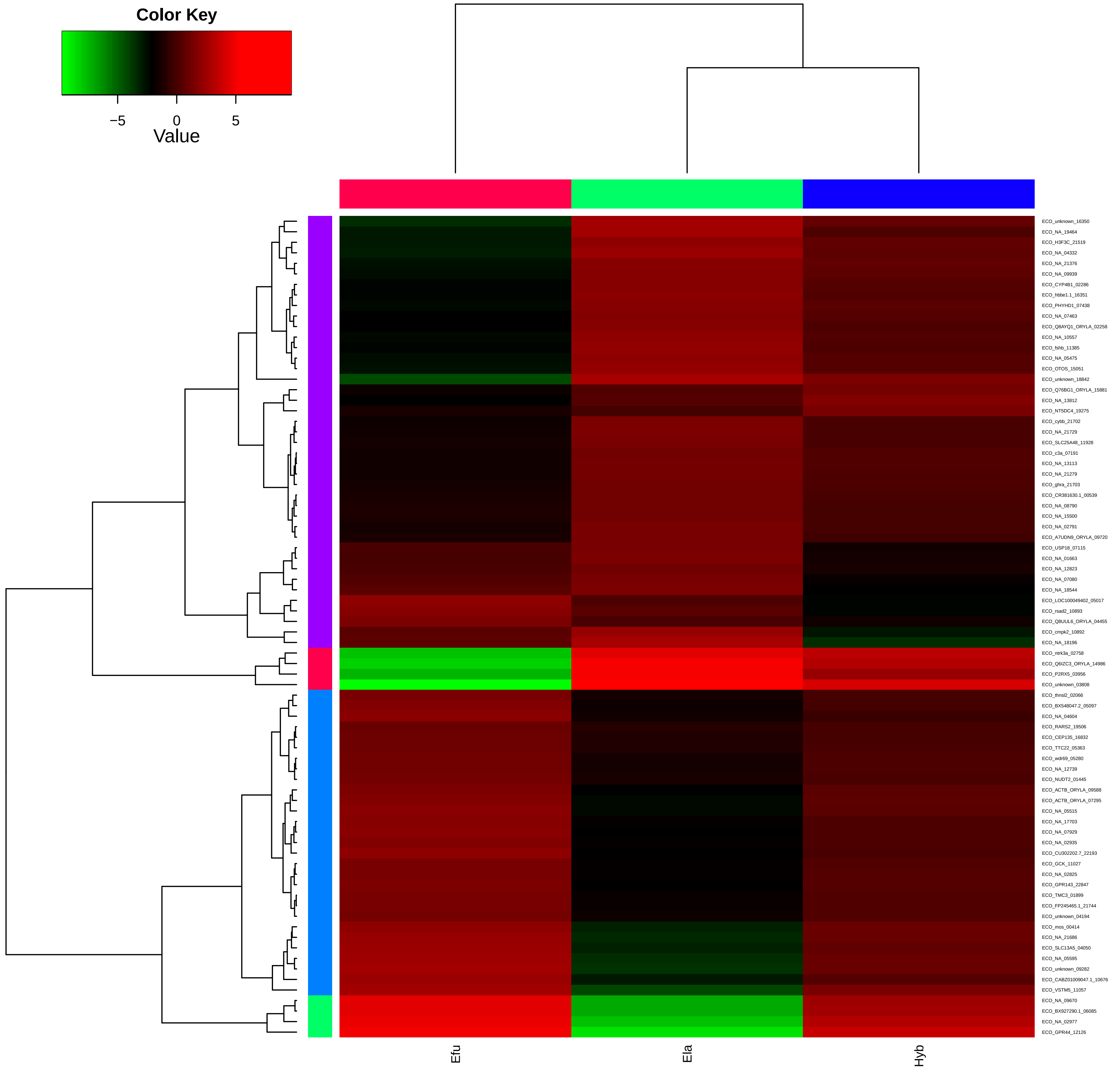

Color Key
−5
0
5
Value
ECO_unknown_16350
ECO_NA_19464
ECO_H3F3C_21519
ECO_NA_04332
ECO_NA_21376
ECO_NA_09939
ECO_CYP4B1_02286
ECO_hbbe1.1_16351
ECO_PHYHD1_07438
ECO_NA_07463
ECO_Q8AYQ1_ORYLA_02258
ECO_NA_10557
ECO_fshb_11385
ECO_NA_05475
ECO_OTOS_15051
ECO_unknown_18842
ECO_Q76BG1_ORYLA_15881
ECO_NA_13812
ECO_NT5DC4_19275
ECO_cybb_21702
ECO_NA_21729
ECO_SLC25A48_11928
ECO_c3a_07191
ECO_NA_13113
ECO_NA_21279
ECO_ghra_21703
ECO_CR381630.1_00539
ECO_NA_08790
ECO_NA_15500
ECO_NA_02791
ECO_A7UDN9_ORYLA_09720
ECO_USP18_07115
ECO_NA_01663
ECO_NA_12823
ECO_NA_07080
ECO_NA_18544
ECO_LOC100049402_05017
ECO_rsad2_10893
ECO_Q8UUL6_ORYLA_04455
ECO_cmpk2_10892
ECO_NA_18196
ECO_ntrk3a_02758
ECO_Q6IZC3_ORYLA_14986
ECO_P2RX5_03956
ECO_unknown_03808
ECO_thnsl2_02066
ECO_BX548047.2_05097
ECO_NA_04604
ECO_RARS2_19506
ECO_CEP135_16832
ECO_TTC22_05363
ECO_wdr69_05280
ECO_NA_12739
ECO_NUDT2_01445
ECO_ACTB_ORYLA_09588
ECO_ACTB_ORYLA_07295
ECO_NA_05515
ECO_NA_17703
ECO_NA_07929
ECO_NA_02935
ECO_CU302202.7_22193
ECO_GCK_11027
ECO_NA_02825
ECO_GPR143_22847
ECO_TMC3_01899
ECO_FP245465.1_21744
ECO_unknown_04194
ECO_mos_00414
ECO_NA_21686
ECO_SLC13A5_04050
ECO_NA_05595
ECO_unknown_09282
ECO_CABZ01009047.1_10676
ECO_VSTM5_11057
ECO_NA_09670
ECO_BX927290.1_06085
ECO_NA_02977
ECO_GPR44_12126
Efu
Ela
Hyb
