# Supplementary figures and images for: Transcriptome analysis reveals the molecular mechanisms underlying growth superiority in a novel grouper hybrid (Epinephelus fuscogutatus♀ × E. lanceolatus♂)
Source: BMC Genet. 2016 Jan 19;17:24. doi: 10.1186/s12863-016-0328-y (PMC4719697; doi:10.1186/s12863-016-0328-y)

Color Key

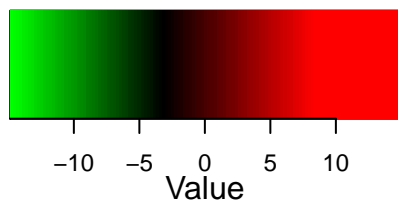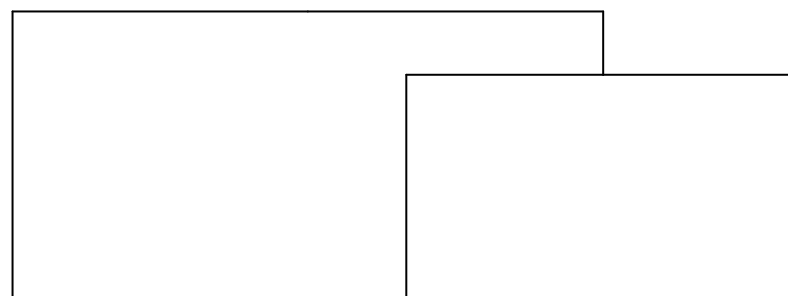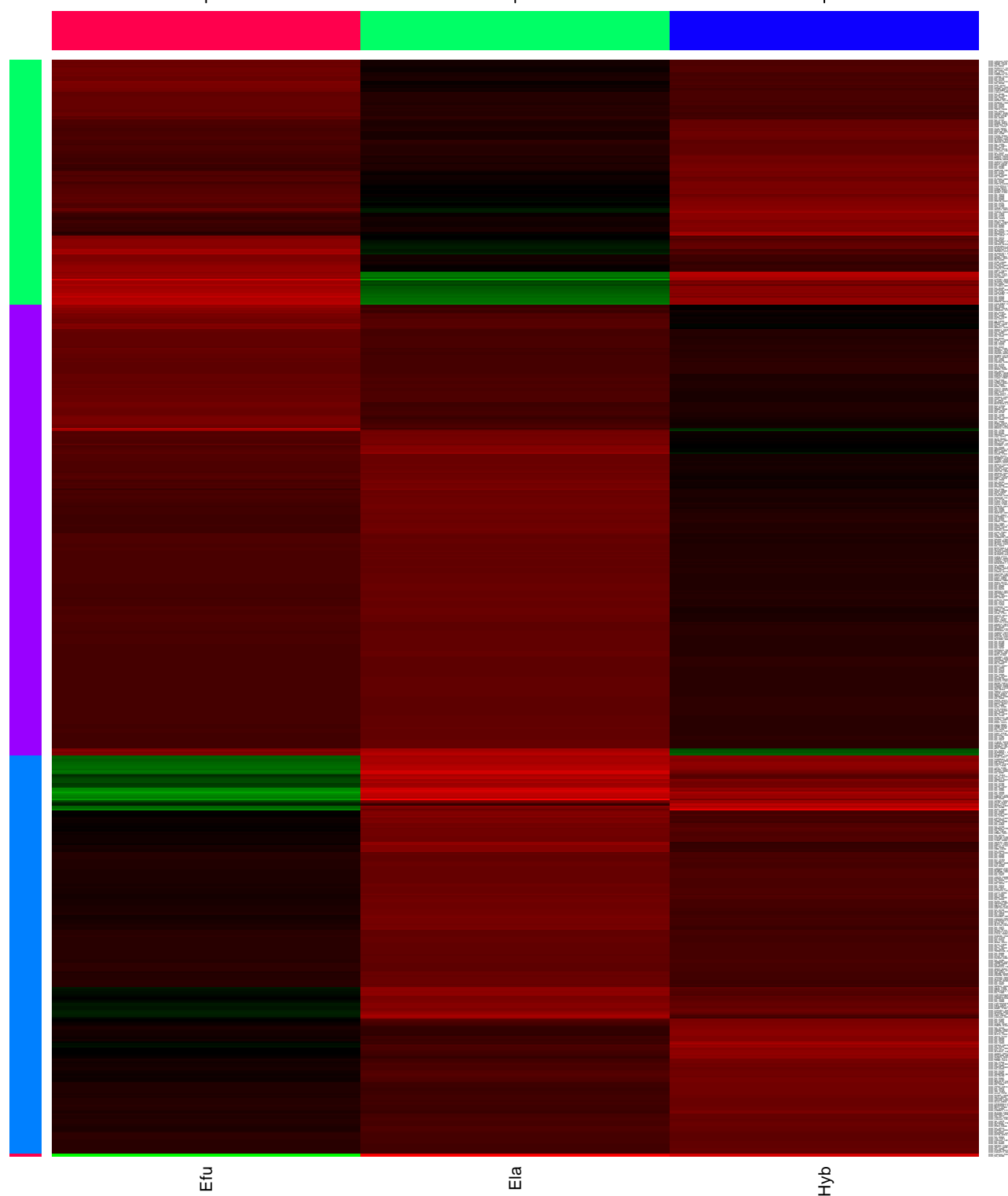

Supplement: Additional file 7: Figure S2. — The hierarchical clustering map of DGEs among three species in the liver. Efu, Ela, and Hyb denote E. fuscoguttatus, E. lanceolatus and their hybrid F1, respectively. (PDF 236 kb) [file 12863_2016_328_MOESM7_ESM.pdf]

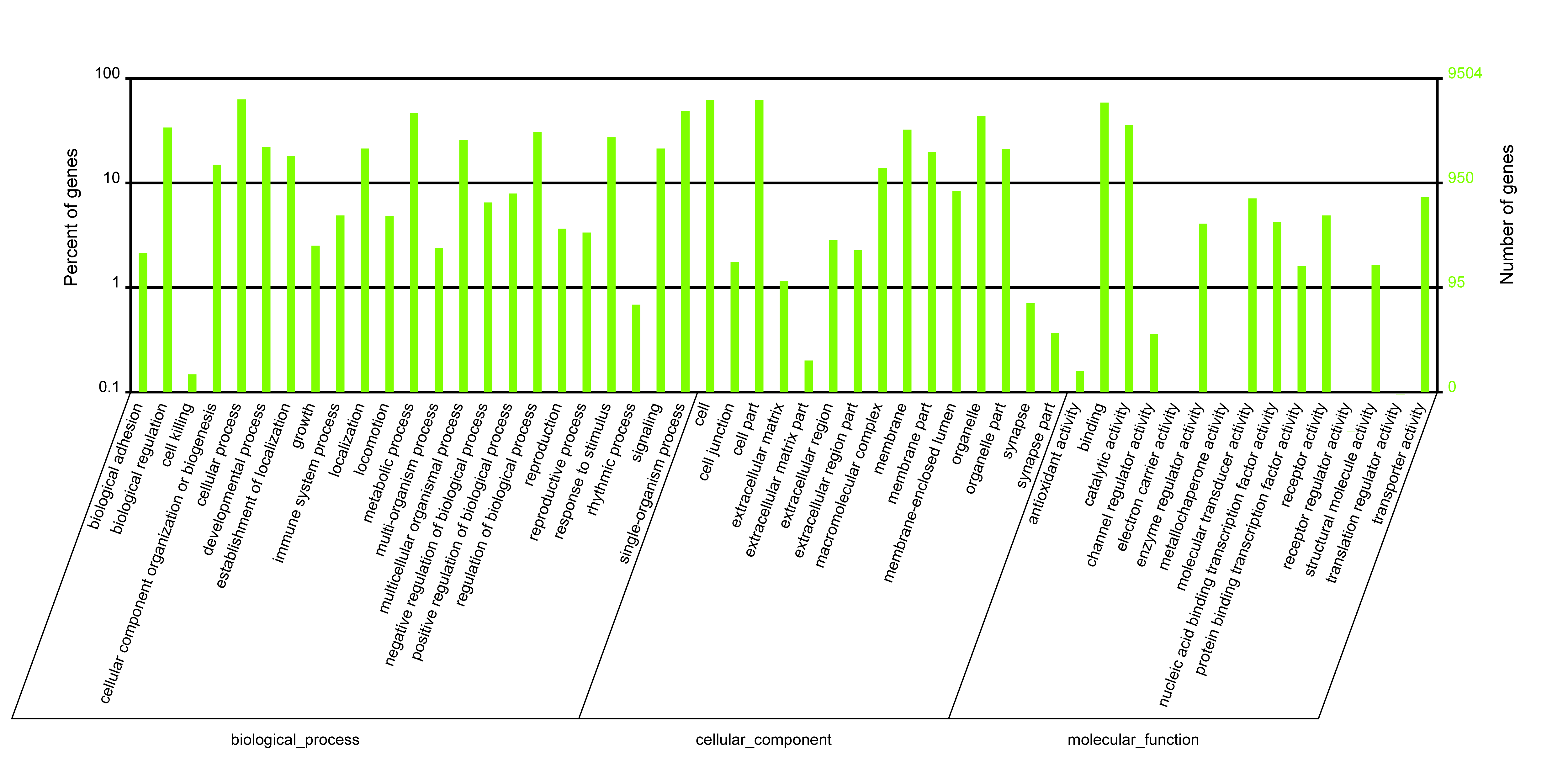

Supplement: Additional file 8: Figure S3. — Functional annotation of grouper transcripts based on GO categorization. The left y-axis indicates the percentage of a specific category of genes in that main category. The right y-axis indicates the number of genes in a category. (TIF 521 kb) [file 12863_2016_328_MOESM8_ESM.tif]
